# Lung cancer screening with low-dose helical CT: results from the National Lung Screening Trial (NLST)

**DOI:** 10.1258/jms.2011.011055

**Published:** 2011-09

**Authors:** Barnett S Kramer, Christine D Berg, Denise R Aberle, Philip C Prorok

**Affiliations:** * Editor-in-Chief, National Cancer Institute Physician Data Query (PDQ) Screening and Prevention Editorial Board, Bethesda, MD, USA; † Division of Cancer Prevention, National Cancer Institute, Bethesda, MD, USA; ‡ Department of Radiological Services, David Geffen School of Medicine, University of California at Los Angeles, Los Angeles, CA, USA; § Division of Cancer Prevention, National Cancer Institute, Bethesda, MD, USA

After long debate about the worth of screening for lung cancer, and even about the merits of doing a randomized trial to address the issue, initial results from the first large-scale randomized controlled trial ever to show a reduction in lung cancer mortality associated with screening were announced to the public last November 4.^1^ The story behind this historical first is informative, and the editor of the *Journal of Medical Screening* played a role in that story. But more of that later.

The National Lung Screening Trial (NLST), launched in September 2002, is a U.S. National Cancer Institute (NCI) sponsored study that is jointly conducted by Lung Screening Study (LSS) screening centres, funded by NCI Division of Cancer Prevention, and the American College of Radiology Imaging Network (ACRIN), funded by the NCI Division of Cancer Treatment and Diagnosis Cancer Imaging Program. A detailed description of the trial overview, rationale and design has been published.^2^ Briefly, 53,454 eligible participants aged 55–74 were recruited by 33 screening centres and were randomly assigned after giving written informed consent to receive either three annual low-dose helical CT scans (LDCT; also called spiral CT by many investigators) or posteroanterior view chest X-rays. The term ‘low-dose’ is used because the average estimated whole-body effective dose in the NLST is 1.5 mSv versus 7 mSv for standard diagnostic chest CT. Because of concerns about the radiation harms associated with CT scans and other medical imaging tests^3–7^, lower doses of radiation are used in the screening setting despite higher resolution achievable with increased doses. (Because the estimated average whole body effective dose of the single posteroanterior chest X-ray is 0.02 mSv, the whole body effective dose ratio of chest X-ray:LDCT:standard diagnostic CT is about 1:75:350.)

Eligibility criteria for the trial included: a ≥30 pack year history of cigarette smoking; smoking cessation ≤15 years if a former smoker; no history of lung cancer; no history of other life threatening cancers in the prior five years; no hemoptysis or weight loss to suggest a diagnosis of lung cancer; and no chest CT in the prior 18 months. The trial had an estimated 90% power to detect a 20% relative reduction in lung cancer mortality. Prior to the launch of the NLST, a feasibility study conducted by six of the LSS screening centres in 3318 former and current smokers established the ability to recruit, randomize and retain volunteers on a study of LDCT versus chest X-ray.^8,9^

Four previous randomized trials of lung cancer screening using chest X-ray with or without sputum cytology led to widespread pessimism about the value of screening for lung cancer.^10–18^ None had shown a reduction in lung cancer mortality, leading to the general perception that any benefits of routine lung cancer screening did not outweigh the harms. However, the trials had insufficient power to detect a reduction in lung cancer mortality. The much larger randomized PLCO (Prostate, Lung, Colorectal and Ovarian) screening trial, designed to have sufficient statistical power, compares chest X-ray with a usual care control arm^19,20^, but has also not shown a benefit to date. The introduction of helical CT represented an advance in technology that triggered renewed enthusiasm for lung cancer screening.^21–27^ Several single arm studies showed a high cancer detection rate with LDCT relative to chest X-ray (as summarized in reference 2).^2^

There were subsequent calls for routine lung cancer screening without waiting for further proof.^28^ There were projections that routine lung cancer screening would be highly cost-effective in terms of life years saved,^28^ but counter-estimates that it would be cost-ineffective^29^ or completely ineffective.^30^ There were even suggestions that doing randomized trials of lung cancer screening with LDCT in the face of the positive single arm studies was unethical.^31,32^ However, trying to prove with confidence that a single arm study demonstrates a reduction in lung cancer mortality is like determining who won a baseball game based on the score of one of the teams. And statistical modelling of what the other team was likely to have scored provides little additional confidence. Powerful confounding factors and systematic biases can muddy interpretation of uncontrolled screening studies.^33^ The NCI accordingly proposed the NLST as a definitive test of the hypothesis generated by the single arm studies. Chest X-ray was chosen as the control arm because it was already being compared with usual care in the PLCO trial.^19,20,34^

There are few issues in medicine that polarize the public, their elected representatives and health professionals more than medical screening. Polarization was evident during preparation for proposing the NLST to the NCI National Cancer Advisory Board. There were arguments, with little evidence, that even conducting a screening trial would leave the impression in the minds of smokers that they need not stop smoking. There was concern that the cost of a randomized trial would siphon money from more basic investigator-initiated research projects. Finally, some were already convinced that the well-known confounders (e.g. healthy volunteer effect, lead time bias and length-biased sampling) could not account for projections from some of the single arm studies that LDCT would decrease lung cancer mortality by at least 80%. And this is where the editor of the *Journal of Medical Screening* enters the story. Professor Wald was asked to review the merits of the trial and to give an independent assessment to the National Cancer Advisory Board. He made the case that a large randomized trial of LDCT screening for lung cancer with lung cancer mortality endpoints would be feasible, affordable and should be instituted quickly. His endorsement of the need for the trial played a crucial role in the decision to fund the trial.

The NLST has been a success. Accrual was brisk, reaching its target in April 2004. Compliance was high: 98.5%, 94.0% and 92.9% in the three successive LDCT screens; 97.4%, 91.3% and 89.5% in the three chest X-ray screens. At its sixth planned interim analysis in October 2010, the independent Data and Safety Monitoring Board unanimously recommended that the results be announced to the public because the primary endpoint had been achieved: a statistically significant 20.3% relative reduction in lung cancer mortality in the LDCT arm. This represented an absolute lung cancer mortality rate of 247 versus 309 per 100,000 person years with LDCT versus chest X-ray. By comparison, the PLCO study, has shown no impact to date of chest X-ray on lung cancer mortality either in the entire PLCO population or in trial participants that met the eligibility criteria for the NLST. Perhaps because lung cancer has such a strong force of mortality in smokers and former smokers, the reduction in lung cancer mortality was accompanied by a statistically significant 6.7% relative reduction in all-cause mortality (1121 versus 1202 deaths per 100,000 person-years). The number of lung cancers diagnosed was 649 and 279 respectively. While it is too early to make an accurate estimate, some of this excess may represent overdiagnosis compared with chest X-ray – the detection of non-lethal tumors that would not have surfaced had it not been for screening. In addition, cumulative screen positivity rates were 24.2% versus 6.9% in the LDCT and chest X-ray, representing an added burden of follow-up associated with LDCT. Although the consequences of the follow-up of these tests have not yet been fully analyzed, the LSS feasibility study showed that LDCT and chest X-ray triggered invasive follow-up procedures in 7% and 4% of participants who had a false positive test.^35^

The NLST has provided a powerful answer to some of the most contentious questions swirling around the issue of lung cancer screening when Professor Wald assessed the state of the evidence in 1999. It is clear that the efficacy of LDCT screening for lung cancer is not as large as the most optimistic estimates from the single arm studies, nor as bad as the statistical models predicting no benefit. Such is the power of a randomized controlled trial (RCT). The NLST has been described as ‘the best umpire in town’ to help decide whether CT screening represents a home run or foul ball.^36^ However, it has not answered all of the important questions that are critical to public health policy decisions. There are remaining issues to address, including the effect on quality of life and adverse effects of treating overdiagnosed cancers. In this regard, the NLST has collected lung pathology specimens that may allow a deeper understanding of the molecular processes that underpin variations in biologic behavior of the cancers. There are also planned and ongoing formal studies of quality of life and cost-effectiveness, as well as modelling studies addressing optimal frequency of screening and age range of optimal effectiveness. Questions will still remain about whether the balance of benefits and harms found in a study conducted by centres that have strong expertise in screening can be maintained in less regulated and less quality-controlled settings in the general community.

Undoubtedly, gaps in our knowledge will also be filled in by other ongoing randomized clinical trials, such as the Danish Randomized Lung Cancer CT Screening Trial^37^, the Detection and Screening of Early Lung Cancer (DANTE) trial^38^, the NELSON trial^39^, the ITALUNG study^40^, the LUSI trial in Germany^41^, and the U.K. Lung Screen (UKLS).^42^ However the aggregate results turn out, we are well-served by randomized controlled trials to provide high-level evidence to inform decisions about whether to institute widespread lung cancer screening as a matter of public health policy. As pointed out by Dr. Robert Young, the chairperson of the NLST Oversight Committee, randomized trials are the best antidote available for medical controversy.^43^

**Note:** Opinions expressed in this manuscript represent those of the authors and do not necessarily represent official opinions or positions of the federal government of the United States or the Department of Health and Human Services.
